# Prognostic model of ubiquitination-related genes in ovarian cancer based on transcriptomic analysis and experimental validation

**DOI:** 10.3389/fimmu.2025.1654180

**Published:** 2025-09-08

**Authors:** Xiaojing Lin, Shu Zhao, Licheng Li, Yuying Huang, Qiang Zhong, Huali Luo, Qizhu Zhang, Shuxiong Xu, Qinshan Li, Mengxing Li

**Affiliations:** ^1^ Department of Obstetrics and Gynecology, Institute of Precision Medicine of Guizhou Province, Affiliated Hospital of Guizhou Medical University, Guiyang, Guizhou, China; ^2^ Department of Clinical Biochemistry, School of Medical Laboratory Science, Guizhou Medical University, Guiyang, Guizhou, China; ^3^ Department of Gynecology and Obstetrics, School of Clinical Medicine, Guizhou Medical University, Guiyang, China; ^4^ Department of Obstetrics and Gynecology, Affiliated Hospital of Guizhou Medical University, Guiyang, Guizhou, China; ^5^ Department of Obstetrics, Guizhou Provincial People’s Hospital, Guiyang, Guizhou, China; ^6^ Department of Hematology, Guizhou Province Institute of Hematology, Guizhou Province Laboratory of Hematopoietic Stem Cell Transplantation Centre, Affiliated Hospital of Guizhou Medical University, Guiyang, Guizhou, China; ^7^ Department of Gynecology, Guizhou Hospital of The First Affiliated Hospital, Sun Yat-sen University, Guiyang, Guizhou, China; ^8^ Department of Urology, Guizhou Provincial People’s Hospital, Guiyang, Guizhou, China

**Keywords:** ovarian cancer, ubiquitination, prognostic model, immunotherapy, FBXO45, Wnt/β-catenin signaling pathway

## Abstract

**Objective:**

Ubiquitination plays a crucial role in the malignant progression of ovarian cancer. With the advent of proteolysis-targeting chimeras (PROTACs) targeting ubiquitin enzymes, precision therapies are now possible. Therefore, it is imperative to ascertain the prognostic significance of ubiquitination-related genes in ovarian cancer.

**Methods:**

A prognostic model based on ubiquitination-related genes was developed using data from TCGA and GTEx databases. Performance was assessed via Kaplan-Meier, ROC curves, and Cox regression; a nomogram was created. The model’s stability was checked using training and test sets. FBXO45 was also experimentally validated in ovarian cancer.

**Results:**

The model, based on 17 genes related to ubiquitination, showed high performance (1-year AUC = 0.703, 3-year AUC = 0.704, 5-year AUC = 0.705). The high-risk group had significantly lower overall survival (P < 0.05). Immune analysis showed higher levels of CD8+ T (P < 0.05), M1 (P < 0.01) and follicular (P < 0.05) cells in the low-risk group. High-risk patients had more mutations in MUC17 and LRRK2, while low-risk patients had more RYR2 mutations. FBXO45 is a key E3 ubiquitin ligase in ovarian cancer, promoting growth, spread and migration via the Wnt/β-catenin pathway.

**Conclusion:**

Ubiquitination-related markers provide reliable prognostic insights and reflect the immune microenvironment in ovarian cancer, offering a basis for clinical targeting strategies.

## 1 Introduction

Ovarian cancer is the leading cause of gynecological cancer-related mortality. In 2018, it accounted for 4.4% of all cancer-related deaths, rising to 4.7% in 2020 (1, 2). 70% of cases are already advanced at diagnosis, with a 5-year survival rate of just 29% (3, 4). The high mortality is compounded by the tumor’s resistance to both chemotherapy and targeted therapies, driven by genetic and epigenetic alterations as well as a complex Tumor Microenvironment (TME). This resistance extends to alternative treatment options (5), making OV a persistent challenge in oncology. As a result, identifying prognostic factors and novel biomarkers for targeted therapeutic strategies has become an urgent clinical priority.

Ubiquitination is the process by which ubiquitin is covalently linked to a substrate, thereby modifying the substrate for either degradation or stabilization. This modification is ordinarily brought about by the interplay of E1 ubiquitin-activating enzymes, E2 ubiquitin-conjugating enzymes, and E3 ubiquitin ligases (6). These enzymes are critical in regulating cellular processes, including tumor proliferation, invasion, apoptosis, DNA damage response, repair mechanisms, metabolism, immune responses, and drug resistance (7–9).

Studies have established a link between ubiquitination-related factors and diverse facets of cancer biology, encompassing tumor initiation, invasion, metastasis, drug resistance, and immune microenvironment modulation. Notable factors include RNF168, UBR5, and WWP2 (10–12). Among these, pathogenic mutations in the ubiquitin ligase BRCA1 (OR, 75.6; 95% CI, 31.6-180.6) elevate the risk of OV by 75-fold (13). To date, 50 ubiquitination-related genes have been targeted by Proteolysis Targeting Chimeras (PROTACs), with several emerging as promising clinical drug targets for cancer treatment (14, 15). Li et al. emphasized that PROTACs offered significant advantages, such as reducing drug dosage and administration frequency, enhancing therapeutic duration, minimizing toxicity, and overcoming drug resistance—making them a promising avenue for future drug development (16). Despite these advancements, the precise role of various ubiquitination-related factors in OV remains poorly understood. Further systematic investigations are required to unravel the molecular mechanisms underpinning their involvement in OV.

This study examined the differential expression between OV and normal ovarian tissues from TCGA and GTEX databases. The analysis was further refined by intersecting these datasets with a set of ubiquitination-related genes, resulting in the identification of 162 co-expressed genes. A risk model for prognosis based on 17 ubiquitination-related genes was constructed through COX univariate analysis, LASSO regression, and the DEVIANCE test. The model’s predictive performance was assessed using Kaplan-Meier curves, ROC curves, and nomograms. External validation was performed by applying the model to the GSE165808 and GSE26712 datasets. Furthermore, the study investigated the immune infiltration characteristics and high-frequency mutation gene distribution patterns of patients in different risk groups. In order to further evaluate the prognostic value of the OV risk model, the biological function of its key component, the ubiquitin ligase FBXO45, was analyzed in the context of OV, and its role in the Wnt/β-catenin pathway was explored. Collectively, these results imply that ubiquitination-related risk models provide useful predictive information about OV patients and could lead to the creation of innovative target-based treatments.

## 2 Materials and methods

### 2.1 Reagents list

The main laboratory reagents and sources are detailed in **Table 1**.

**Table 1:** Main laboratory reagents and sources.

Reagents	Sources
DMEM (12800017)	Gibco
RNAiso Reagent (9108)	Takara
Real-time fluorescence quantitative PCR kit (RR064A)	Takara
Trypsin (03-050-1A/03-050-1B)	BI
RNA Reverse Transcription Kit (RR037A)	Takara
Lipo2000 transfection reagent (31985-062)	Thermo fisher
FBS (04-001AUS-1A/)	BI
5× Protein loading Buffer (P104)	Solarbio
High-performance RIPA lysate (R0010)	Solarbio
Phosphatase/Protease Inhibitor Mix (P1081)	Beyotime
ECL Chemiluminescent Liquid (WBKLS0500-2)	Millipore
TEMED (G4829)	Solarbio
4% paraformaldehyde (DF0133)	Leagene
1% crystal violet staining solution (G106)	Solarbio
GAPDH (5174S)	CST
FBXO45 (NM_001105573)	Abmart
WNT1 (WL05209)	Wanleibio
β-cadherin (51067-2-AP)	Proteintech
C-myc (WL01781)	Wanleibio

### 2.2 Data collection and processing

376 tumor and 88 normal ovarian tissue samples’ transcriptomes and clinical profiles were accessed via the TCGA-OV (https://www.cancer.gov/) and GTEx databases (UCSC Xena, https://xenabrowser.net). These datasets were subsequently used for evaluation as part of the training set. Using the ‘edgeR’ package (17). The differential gene expression between OV and normal tissues was examined. For OV, differentially expressed genes (DEGs) were identified by applying a |logFC| ≥ 1 and a corrected p-value threshold of < 0.01. GSE165808 and GSE26712 datasets were used to validate the prognostic model. These datasets include 49 and 153 OV samples with survival data.

### 2.3 Candidate gene screening

The list of ubiquitinating enzyme UBQ genes was derived from the UUCD (http://uucd.biocuckoo.org/) (downloaded March 2017) and then modified by removing non-UBQ genes. The final UBQ genome included 929 genes, grouped into the established UUCD categories: E1 (8 genes), E2 (39 genes) and E3 (882 genes). A Venn diagram was used to intersect these genes with DEGs, identify 162 co-expressed genes. COX analysis selected ubiquitination-related genes with a P value < 0.05, identifying the top 20 genes associated with OV survival prognosis.

### 2.4 Predictive model construction

LASSO regression analysis and the DEVIANCE (18) test were applied to the candidate genes, with a selection criterion of |logFC| ≥ 1 and adjusted *p*-values < 0.05. The prognostic model was constructed using 17 genes. The risk score was calculated by


Risk score=∑n i Coef i × Ai

Coef_i = regression coefficient; A_i = gene expression level (19). Patients were grouped by risk (high/low) using the median risk score. Performance of the model was evaluated using survival analysis and ROC curve analysis.

### 2.5 Immune landscape and gene mutation analyses

The risk model uses expression profiles of 17 genes to assess 22 immune cell levels with R package e1071. First, stromal and immune scores were calculated and visualized using ESTIMATE (20). The Wilcoxon rank sum test was employed to analyze the differences between the two groups. Furthermore, the TCGA database was searched for single nucleotide variation (SNV) data for OV, and gene mutation analysis was performed using the “maftools” software package (21) based on somatic mutation data, focusing on genes with higher mutation frequencies in the two patient groups and using a waterfall diagram to show.

### 2.6 Single-cell RNAseq analysis

Single-cell RNA sequencing data of ovarian cancer were obtained from the E-MTAB-8381 dataset in ArrayExpress. Cells exhibiting a gene count of fewer than 200, or a mitochondrial gene count that exceeds 15%, are to be considered as having undergone a significant deviation from the standard. The process of gene expression was eliminated. Genes that exhibited expression in fewer than three cells were excluded from further analysis. The data underwent a process of normalization, employing the LogNormalize method. This was followed by the identification of 2,000 genes that exhibited high variability, along with scaling and principal component analysis (PCA). The top 20 components were used for graph-based clustering and UMAP reduction. Cell type annotation was conducted using canonical marker genes and automated classification via the “SingleR” package, referencing the Human Primary Cell Atlas (https://www.proteinatlas.org/ENSG00000174013-FBXO45/single+cell/ovary).

### 2.7 Cell culture and transfection

Human OV cell lines A2780 and HEY were obtained from the Cell Center, Institute of Basic Medical Sciences, Chinese Academy of Medical Sciences. Cell validation was performed via short tandem repeat (STR) analysis, and mycoplasma testing yielded negative results. DMEM and RPMI 1640 media, along with fetal bovine serum, were sourced from Gibco (USA). Penicillin-streptomycin solution (1%) was procured from Wuhan Boster Biological Technology Co., Ltd. Primary and secondary antibodies for Western blot analysis, as well as the corresponding diluents, were obtained from Wuhan Boster Biological Technology Co., Ltd. The transfection reagent Lipo8000™ was purchased from Shanghai Beyotime Co., Ltd. FBXO45-specific small interfering RNAs (si-FBXO45) and control siRNA (NC) were synthesized by Shanghai Sangon. Horseradish peroxidase-labelled goat anti-rabbit IgG was supplied by Wuhan Sanying Biotechnology Co., Ltd. The CCK-8 cell proliferation assay kit was purchased from MedChemExpress (USA). Matrigel matrix was sourced from Corning (USA), and Trizol reagent (T9424, 200 ml) was obtained from Sigma (USA). qPCR reagents, including SYBR^®^ Premix Ex-Taq™ II (Tli RNaseH Plus, RR820Q), were provided by Takara (Japan). The target sequences for FBXO45 siRNA were listed in **Table 2**.

**Table 2:** Three target sequences of FBXO45 small interfering RNA (siRNA).

Si1- FBXO45	UUAAUGUAGACAUUCCUGGT
Si2- FBXO45	UAGUAGAUUAUUGUCCACCT
Si3 -FBXO45	UAAACCAAAGUCACUUCUGT

### 2.8 Western blotting

The same RIPA lysis buffer was used for all samples of the WB experiment and quantified using BCA method after extraction, and each sample was repeated three times, and based on the quantification results, all samples were adjusted to the same concentration of 10 μg/μL with the lysis buffer, and the upper volume of about 5 μL corresponded to 50 μg of total protein. Western Blot quantification was performed by ImageJ (NIH version 1.53) analysis. After all images were converted to 8-bit, the bands were delineated with the rectangle tool, the background was subtracted, and the integrated density was recorded. Target protein expression was normalized by an internal reference protein (GAPDH), and cross-gel experiments were corrected by internal control samples. Data are from 3 independent experiments and are expressed as mean ± SD. Exposure time was controlled within 30 s to avoid signal saturation. The membranes were incubated with primary antibodies (FBXO45, 1:1000; WNT1 1:1000; β-cadherin, 1:1000; C-myc, 1:1000; GAPDH, 1:50,000) then HRP-conjugated anti-rabbit secondary antibody (1:5000) was added. Protein bands were visualized using ECL reagent (Bioworld, Nanjing, China).

### 2.9 Enrichment analyses

We followed the methods of Chen et al. (22). Enrichment analyses were performed using the “ClusterProfiler” R package. Gene Set Enrichment Analysis (GSEA) and OverRepresentation Analysis (ORA) were both conducted in order to investigate the biological pathways and processes associated with the gene expression profiles. It is important to note that all enrichment analyses gave rise to adjusted p-values (Benjamini-Hochberg corrected) of <0.05, which have been considered to be statistically significant. Pathway visualization and interpretation were aided by enrichment maps, dot plots, and ridge plots generated via ClusterProfiler or associated visualization functions.

### 2.10 Statistical analysis

R software (version 4.2.0; New York, USA) was used for data analysis and visualization. The Student’s t-test compares normally distributed quantitative data, the Wilcoxon test for non-normally distributed data between groups. Significant levels: * P < 0.05, ** P < 0.01, *** P < 0.001. For detailed methods, in **Supplementary Material 1**.

## 3 Results

### 3.1 Development of a prognostic model for ubiquitination-related genes and internal validation

**Figure 1:**
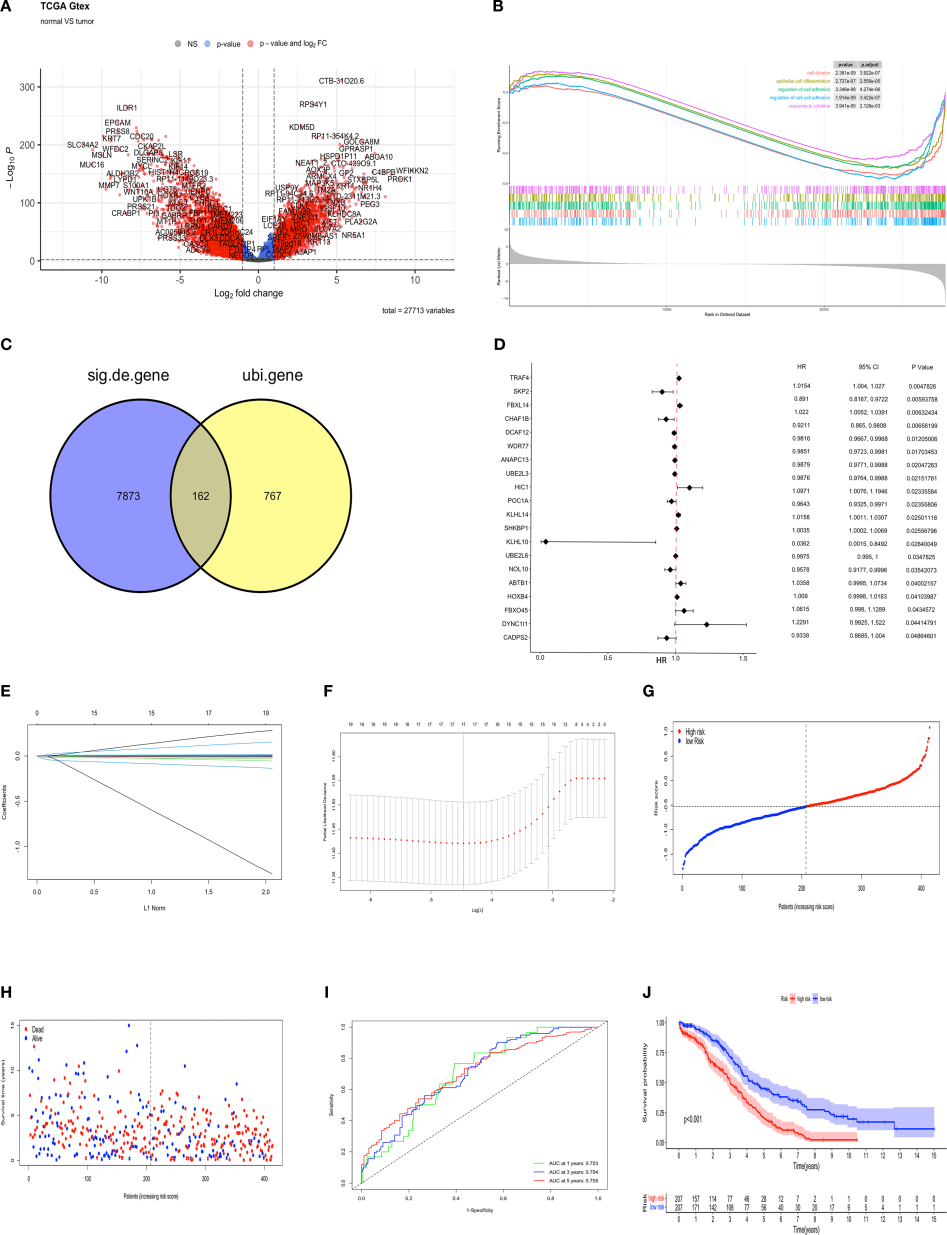
Development of a prognostic model for ubiquitination-related genes and internal validation. **(A)** Volcano plot of up-regulated (n = 3516) and down-regulated (n = 4519) genes (FDR < 0.01, |LogFC| > 1) between OV and normal tissues. **(B)** GSEA of KEGG pathways for DEGs in high/low expression OV groups. **(C)** The Venn diagram illustrating the common features of DEGs and ubiquitination-related genes (n=162). **(D)** Forest plot from univariate Cox analysis. **(E)** LASSO regression coefficients for ubiquitination-related genes, with each curve representing a gene associated with ubiquitin. **(F)** Parameter selection process in the LASSO model. **(G)** The training set contains risk scores. **(H)** Training set survival status. **(I)** ROC curves for predicting 1- to 5-year OS in the training set. **(J)** K-M survival curves for high- and low-risk groups in the training set.

The identification of prognostic factors for OV was facilitated by data obtained from the TCGA-OV and GTEx databases. Clinical and transcriptomic (HTSeq-FPKM) data from 376 OV tissues and 88 normal ovarian tissues were downloaded. A total of 8,035 genes were identified that exhibited differential expression (DEGs) between ovarian and normal tissues, including 3,516 upregulated and 4,519 downregulated genes (p < 0.01, |log2FC| > 1) (**Figure 1A**). GSEA analysis was performed based on the differential expression results between tumor and normal tissues. In tumor tissues, pathways such as “cell division,” “epithelial cell differentiation,” “regulation of cell adhesion,” “regulation of cell -cell adhesion,”and “cytokine response” were found to be suppressed (**Figure 1B**). The NES values of the five pathways are respectively: -1.76,-1.59,-1.63,-1.77,-1.46. Venn analysis was used to identify the intersection between differentially expressed genes and 929 ubiquitination-related genes reported in the literature (23). These ubiquitination genes contained validated and predicted E1, E2 and E3 enzymes and adapters. Ultimately, 162 ubiquitination-related genes significantly co-expressed in ovarian cancer tissues were obtained (**Figure 1C**). These 162 genes were further analyzed through COX univariate analysis, leading to the identification of the top 20 ubiquitination-related genes (*p <* 0.05) as candidates associated with OV survival prognosis (**Figure 1D**). The candidate genes underwent LASSO regression analysis and the DEVIANCE test, identifying 17 genes for the establishment of the OV prognostic model (**Figures 1E, F**). The model was implemented in the TCGA database, and the median risk score was utilized to stratify patients in the OV training set into high- and low-risk groups. (**Figures 1G, H**). The model’s predictive accuracy was assessed through the generation of a time-dependent ROC curve. The AUC for OV at 1, 3, and 5 years was 0.703, 0.704, and 0.705, respectively, demonstrating strong predictive performance (**Figure 1I**). The Kaplan-Meier analysis revealed a statistically significant disparity in survival outcomes between the groups, with high-risk patients showing notably worse OS (**Figure 1J**). To further evaluate the model’s clinical applicability, a nomogram was built using the ubiquitination-related score and five clinical features (age, gender and stage) to predict 1-, 3-, and 5-year survival rates. Calibration plots indicated that the model predicted survival rates closely mirrored the actual rates, validating the model’s potential (**Supplementary Figure S2**).

### 3.2 External datasets validate the model’s generalizability

**Figure 2:**
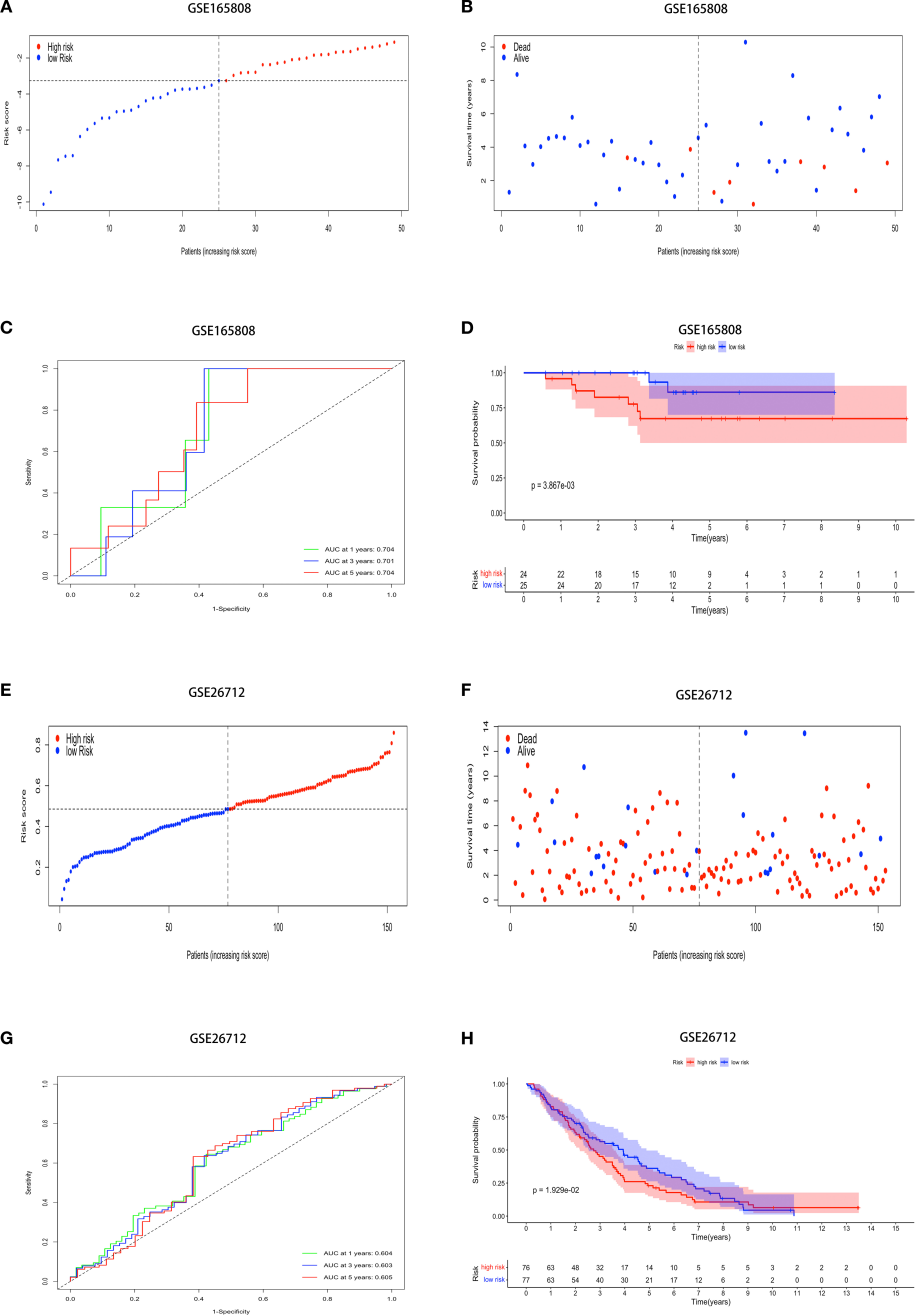
External datasets evaluate the model’s generalizability. Demonstrating stable prediction performance across diverse datasets. The risk score, survival status, ROC curves, and K-M survival curves in the **(A-D)** GSE165808, and **(E-H)** GSE26712.

The model was validated using external datasets GSE165808 and GSE26712. Patient risk scores were calculated and classified (**Figures 2A, E**). The dataset revealed a clear difference between groups (**Figures 2B, F**). ROC curve analysis of the model’s performance in OV across both datasets revealed the following AUCs for one-year, three-year, and five-year predictions: (0.704, 0.701, and 0.704) and (0.604, 0.603, and 0.605) (**Figures 2C, G**). Kaplan-Meier analysis showed obvious prognostic differences between groups, with high-risk patients exhibiting notably lower overall survival (OS) compared to their low-risk counterparts (p < 0.01, and p < 0.05) (**Figures 2D, H**).

### 3.3 Immune characteristics of the prognostic model in the OV microenvironment

**Figure 3:**
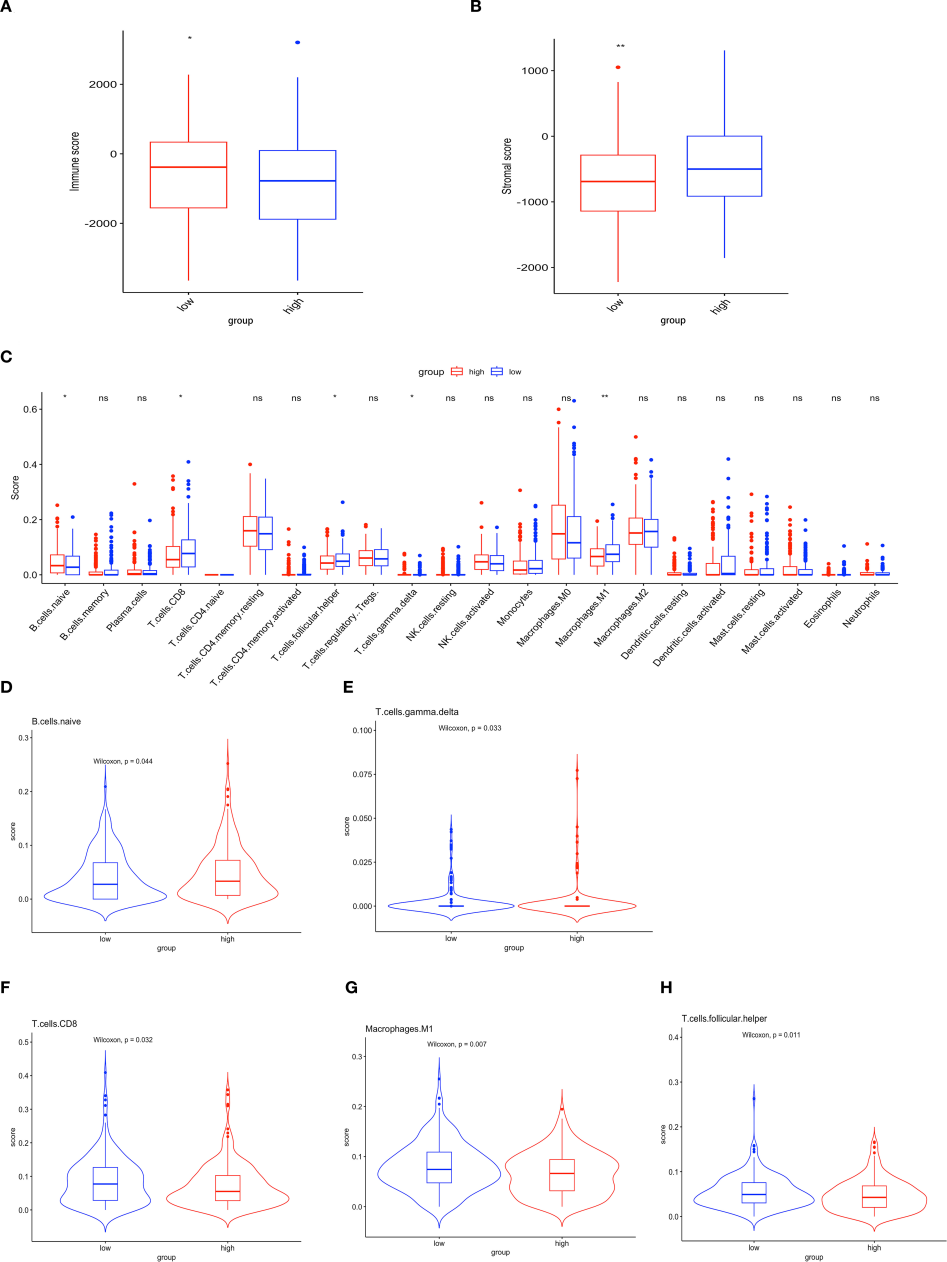
Immune characteristics of the prognostic model in the OV microenvironment. **(A)** Immune scores. **(B)** Stromal scores. **(C)** Histogram illustrating the differential expression of tumor-infiltrating immune cells between the high-risk and low-risk groups. Red and blue represent high- and low-risk groups, respectively (ns p>0.05; *p<0.05; **p<0.01). **(D-H)** Comparative analysis of the TME between the two groups.

To explore the immune landscape associated with the prognostic model, first, we used ESTIMATE to obtain stromal and immune scores for each sample, with the immune scores in the high-risk group being observed to be lower than those in the low-risk group (**Figure 3A**) and higher stromal S score was observed in patients classified as low-risk (**Figure 3B**), and the ORT algorithm to assess immune cell infiltration in both groups (**Figure 3C**). The analysis demonstrated a negative correlation between immune infiltration, encompassing both naïve B cells and γδT cells, and the score within the low-risk group (p < 0.05) (**Figures 3D, E**). Conversely, patients in the low-risk group exhibited elevated levels of CD8+ T cells (p < 0.05), M1 macrophages (p < 0.01), and Tfh cells (p < 0.05) (**Figures 3F-H**). The results indicate a correlation between ubiquitination-associated scores and the extent of tumor cell infiltration and activity in the OV microenvironment.

### 3.4 Identification of mutational features of the genome

In order to provide further clarification regarding the mechanisms underlying the prognostic risk score’s effectiveness in predicting patient outcomes, the mutation frequency associated with the prognostic model was examined. SNV data for OV were retrieved from the TCGA database. Using the Maftools R package, the mutation profiles of the relevant genes were thoroughly analyzed. The waterfall plots displayed the top 10 most frequently mutated genes in both groups. TP53, TIN, CSMD3, and MUC16 mutations were observed in both groups (**Supplementary Figures S3A, B**), suggesting their potential involvement in OV pathology. Notably, the high-risk group exhibited mutations in MUC17 and LRRK2, while the low-risk group had more RYR2 mutations.

### 3.5 FBXO45 is overexpressed in OV and correlates with poor prognosis

**Figure 4:**
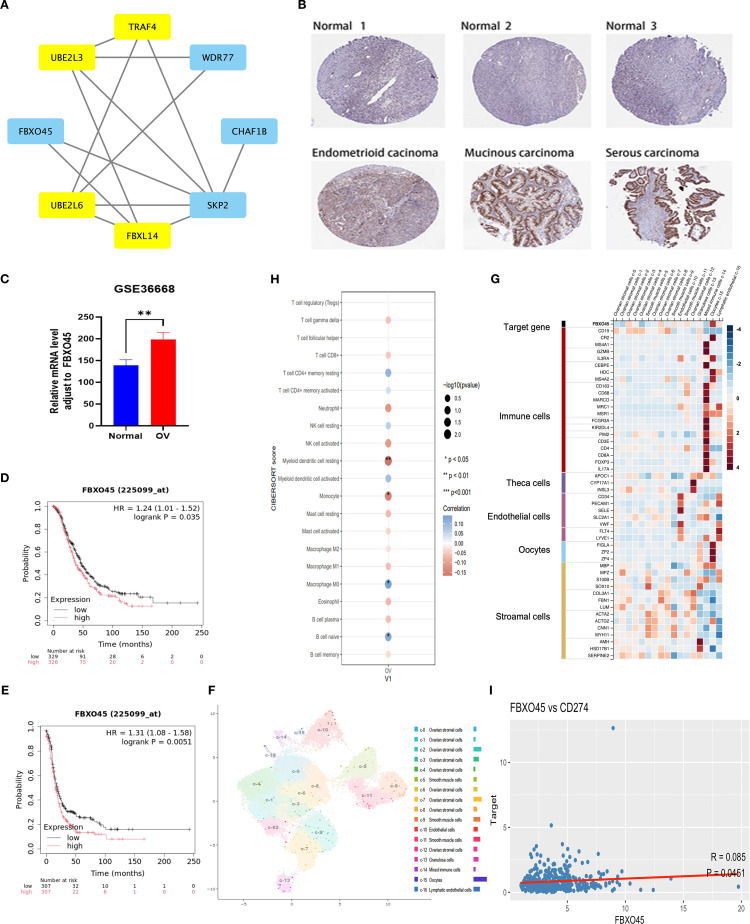
FBXO45 is overexpressed in OV and correlates with poor prognosis. **(A)** PPI network illustrating the interactions among candidate genes. **(B)** FBXO45 expression comparison between normal ovarian tissues and various OV types, based on IHC data from the HPA database. **(C)** FBXO45 is expressed in normal ovarian tissues and plasmacytoid ovarian cancer in the GSE36668 dataset. **(D, E)** PFS and OS analysis for OV patients with FBXO45 involvement, sourced from the R2 genomic analysis platform. **(F)** UMAP plots (left) and histograms (right) depicting RNA expression of the identified single-cell clusters in OV tissues. **(G)** The top 20 markers heat map. **(H)** Immune cell abundance estimation using CIBERSORT. **(I)** Spearman correlation analysis of FBXO45 and CD274 in TCGA.

In order to further elucidate the pivotal function of ubiquitination-related genes in ovarian cancer, significant interactions between the eight core proteins included in the ubiquitination-associated prognostic model were identified by protein interaction network PPI analysis (**Figure 4A**), namely TRAF4, UBE2L3, FBXO45, UBE2L6, FBXL14, SKP2, CHAF1B, WDR77 The protein with the highest impact (HR 1.0615) in univariate regression based on the above proteins was selected for further validation. IHC data from the Human Protein Atlas (https://www.proteinatlas.org/search/FBXO45) confirmed that FBXO45 expression was elevated in ovarian endometroid carcinoma, mucinous carcinoma, and serous carcinoma samples compared to normal ovarian tissues (**Figure 4B**). FBXO45 was highly expressed in plasmacytoid ovarian cancer in the GSE36668 dataset of the GEO database by analyzing data from four normal ovaries and four patients with serous ovarian cancer (**Figure 4C**). Furthermore, analysis using the Kaplan-Meierdatabase (https://kmplot.com/analysis/index.php?P=service&cancer=ovar) demonstrated that higher FBXO45 expression linked to lower PFS and OS in ovarian cancer patients, in contrast to those with low FBXO45 expression (**Figures 4D, E**). UMAP projection was used to visualize the clustering and annotation results. Each point on the graph represents a single cell, colored according to its assigned cell type as determined by marker gene expression. The annotation included stromal cells (expressing MPZ, ACTA2, SERPINE2), immune cells (marked by CD45), oocytes (FIGLA, ZP2), and endothelial cells (CD34, PECAM1), all of which displayed distinct clustering patterns on the UMAP plot (**Figures 4F, G**). Subsequently, the STAR-Counts data and the corresponding clinical information of OV tumors were downloaded from the TCGA database (https://portal.gdc.cancer.gov) and analyzed the immune cell infiltration using CIBERSORT after standardizing the data (**Figure 4H**). The analysis demonstrated a positive correlation between FBXO45 expression and both naïve B cells and M0 macrophages and negatively correlated with monocyte and myeloid dendritic cell dormancy. Immune checkpoint-associated transcripts extracted from OV patients showed that FBXO45 expression was found to correlate positively with PD-L1 (CD274) (**Figure 4I**).

### 3.6 FBXO45 modulates proliferation, invasion, and migration of OV cells

**Figure 5:**
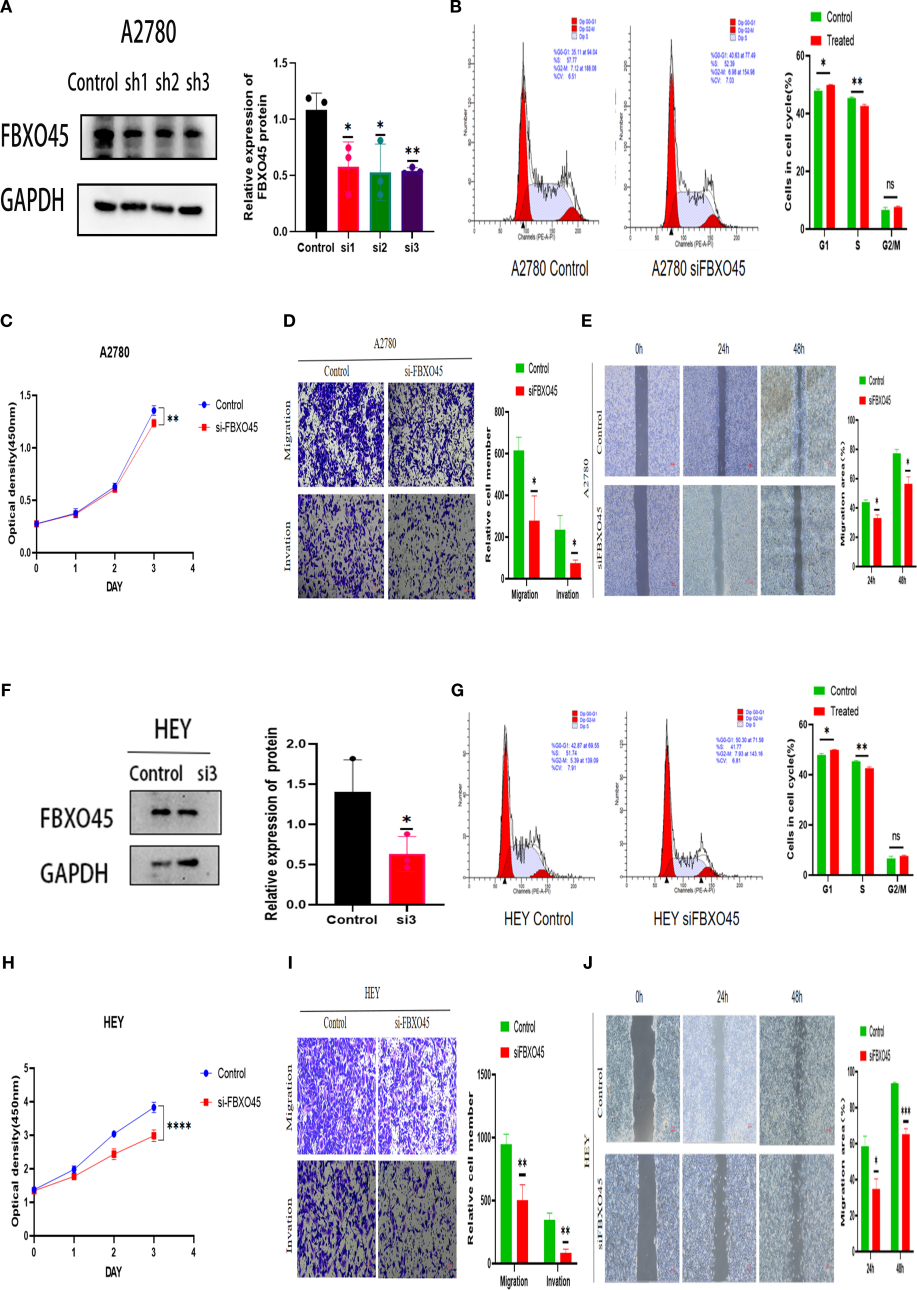
FBXO45 modulates the proliferation, invasion, and migration of OV cells. **(A, F)** Western blotting assessing the efficiency of FBXO45 knockdown in A2780 and HEY cells. **(B, G)** Cell cycle analysis evaluating the proliferative capacity of OV cells following FBXO45 knockdown. **(C, H)** The CCK-8 assay measuring cell proliferation in OV cells post-FBXO45 knockdown (OD450). **(D, I)** Transwell assays evaluating the invasive and migratory potential of OV cells after FBXO45 gene silencing. **(E, J)** Assessing the migration ability of OV cells following FBXO45 knockdown for wound healing assays. (*p <0.05; **p <0.01; ***p <0.001; ****p <0.0001).

The impact of FBXO45 expression on the malignant behavior of OV was assessed by transfecting A2780 and HEY cells with FBXO45-targeting siRNA. Successful knockdown of FBXO45 was confirmed (**Figures 5A, F**), after which the proliferative, invasive, and migratory capacities of OV cells were evaluated. CCK8 and cell cycle assays demonstrated that FBXO45 silencing suppressed OV cell proliferation (**Figures 5B, C, G, H**). Furthermore, transwell and invasion assays revealed a significant reduction in invasion following FBXO45 knockdown (**Figures 5D, I**). Additionally, Wound healing assays also showed a marked decrease in migration ability when FBXO45 was depleted (**Figures 5E, J**). The obtained results indicate that FBXO45 knockdown suppresses the proliferation, migration, and invasion of OV cells.

### 3.7 FBXO45 activates the Wnt/β-catenin pathway

**Figure 6:**
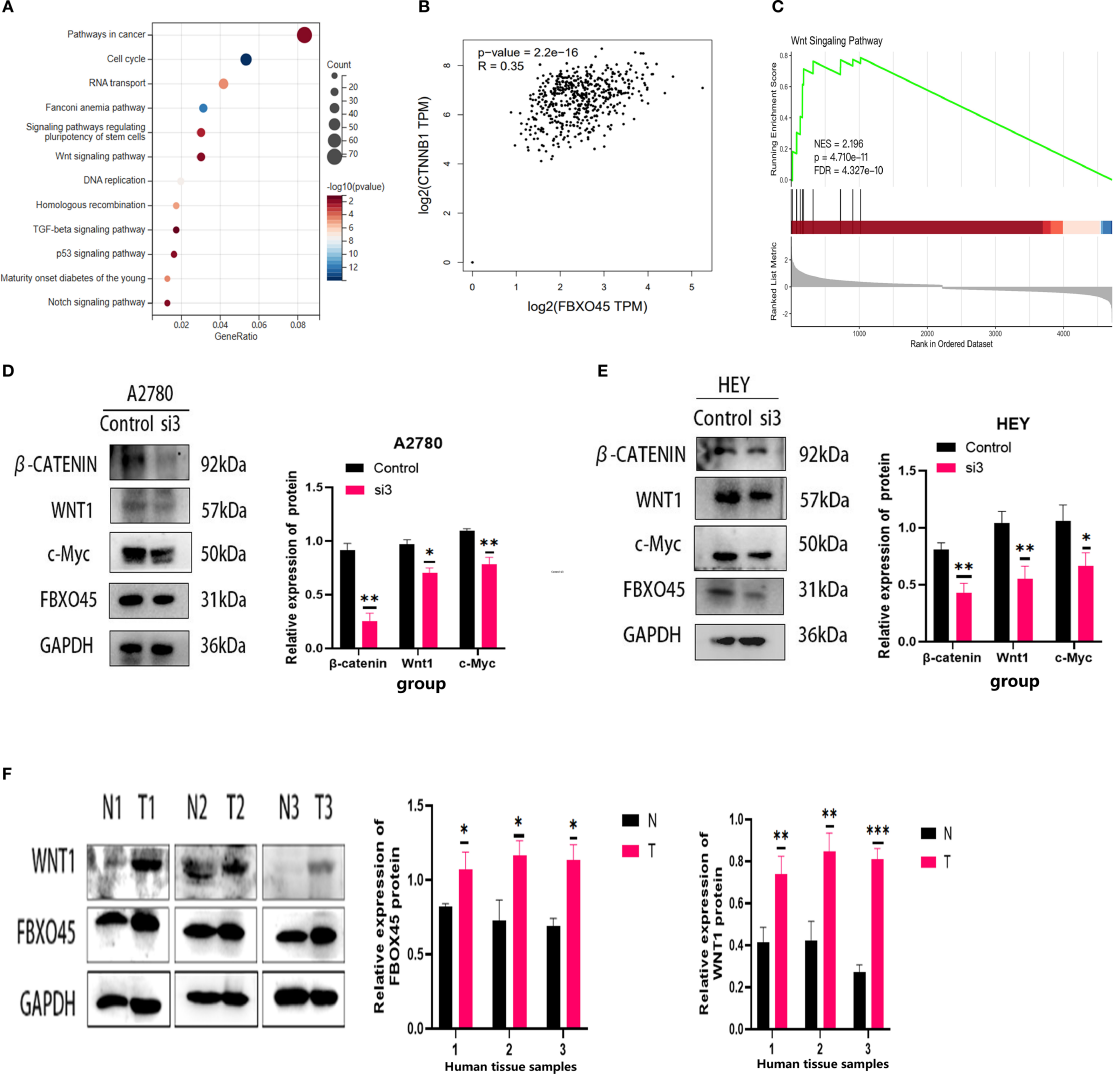
FBXO45 activates the Wnt/β-catenin signaling pathway. **(A)** KEGG enrichment analysis. **(B)** Pearson correlation analysis revealing the relationship between FBXO45 and β-catenin. **(C)** GSEA of the FBXO45 and Wnt/β-catenin signaling pathway. **(D, E)** Western blot analysis assessing the impact of FBXO45 knockdown on key proteins in the Wnt/β-Catenin pathway in A2780 and HEY cells. **(F)** Western blot analysis of FBXO45 and WNT1 expression in three normal ovarian tissues and three OV tissues, accompanied by statistical analysis (*p < 0.05; **p < 0.01; ***p < 0.001).

In order to provide further elucidation on the biological role and mechanism of FBXO45 in OV progression, the samples contained within the TCGA database were categorized into groups of high and low expression, based on the median expression of FBXO45. KEGG enrichment analysis of the high-expression group revealed a significant association of FBXO45 with cancer-related pathways, the cell cycle, RNA transport, and WNT signaling pathways (**Figure 6A**). Further analysis of TCGA RNAseq data using the GEPIA online tool revealed a positive correlation between FBXO45 expression and β-catenin levels in the WNT/β-catenin pathway (**Figure 6B**). Further analysis using GSEA revealed that FBXO45 upregulated this pathway, The NES values of the pathways is 2.196. (**Figure 6C**); Western blotting confirmed that silencing of FBXO45 in A2780 and HEY cells inhibited signaling while suppressing c-Myc expression. (**Figures 6D, E**). Meanwhile, we found that the high expression of FBXO45 in ovarian cancer tissues was accompanied by a corresponding elevation of WNT1 protein in clinical samples of 3 ovarian cancer tissues and normal ovarian tissues (**Figure 6F**). These results lend support to the hypothesis that FBXO45 activates the Wnt/β-catenin signaling pathway, contributing to the malignant development of ovarian cancer.

## 4 Discussion

Despite recent advancements in medicine, the clinical prognosis of OV remains a significant challenge in oncology (24, 25). Ubiquitination modification has been demonstrated to play a critical role in the processes of OV, including tumor progression, treatment resistance, and the TME (26, 27). As research into the role of ubiquitination-related genes in OV has deepened, it has become clear these genes may help with diagnosis and offer new ways to treat the disease.

With the increasing research on the function of ubiquitin-related genes, their clinical value as potential prognostic markers and therapeutic targets has gradually emerged. The present study developed a survival prognostic model for OV patients by means of the identification of differentially expressed ubiquitin-related genes through RNA sequencing. Validation with the training set and two independent cohorts confirmed that the model effectively stratified patients into high- and low-risk groups. The above experimental results demonstrate the stability of prognostic models based on ubiquitination-associated genes have stable predictive effects and good generalization ability in external datasets.

Additionally, Ubiquitination plays a key role in immunomodulation and influences tumor progression by modulating the host immune response. For example, inhibition of USP18 enhances UBCH5- and Nedd4-mediated CSF1R proteasomal degradation, thereby increasing the number of antitumor macrophages within the TME (28). Similarly, PD-L1 is a unique target for UFMylation. Decreased expression of UFL1 has been demonstrated to reduce the UFMylation of PD-L1, thereby stabilizing PD-L1 and imparting immune evasion (29). The study established a correlation between elevated risk scores and a poorer prognosis, as well as immunosuppression. Patients in the low-risk group exhibited higher levels of activated CD8+ T cells, M1-macrophages, and follicular helper T cells within the TME. This may result in a prolonged survival period for patients by enhancing anti-tumor immune responses. Conversely, patients in the high-risk group demonstrated low immunity scores, and stromal low scores exhibited a more pronounced immunosuppressive profile, which may have a deleterious effect on the outlook for high-risk patients. These findings suggest that patients with low risk may demonstrate a heightened sensitivity to immune checkpoint inhibitor therapy.

Among the identified mutations, TP53, TIN, CSMD3, and MUC16 were commonly mutated in both risk groups. MUC17 and LRRK2 mutations were more prevalent in the high-risk group, while RYR2 mutations were more prevalent in the low-risk group. Low MUC17 expression in biliary cancer correlates with poorer survival and vascular invasion (30). LRRK2 inhibits rapid vesicle circulation, thereby promoting a novel signaling axis through the PI3K-Akt immune response to enhance chemokine receptor signal transduction (31). Additionally, a correlation has been demonstrated between low RYR2 expression and unfavorable prognoses in patients diagnosed with thyroid and breast cancer patients (32, 33); however, mutations at these loci remain underexplored in OV. Zibi Marchocki and colleagues identified four gene mutations associated with platinum resistance in OV cases following neoadjuvant treatment, including MUC17 (34). Research on OV has demonstrated that elevated LRRK2 expression can suppress cell proliferation, invasion, and migration (35). Whole exome sequencing of 87 patients with ovarian yolk sac tumors revealed that cancer driver mutations in eight patients with persistent or recurrent disease included ANKRD36, ANKRD62, DNAH8, MUC5B, NUP205, and RYR2 (36). Differences in the distribution of these mutations suggest potential differences in genomic instability between high- and low-risk patients, and mutations in high-risk groups may influence prognosis and treatment outcomes in OV by promoting tumor malignancy and impairing immune function.

The study created a prognostic model using 17 ubiquitin-associated genes and found that eight proteins interacted to form the model’s core. These core genes—TRAF4, UBE2L3, FBXO45, UBE2L6, FBXL14, SKP2, CHAF1B, and WDR77—have been implicated in tumor progression in previous studies.

TRAF4 has been determined to be a prognostic biomarker in OV, with elevated expression levels observed in OV cell lines. TRAF4 silencing has been shown to inhibit cell proliferation, migration and invasion, and stem cell factor expression. Moreover, sh-TRAF4 suppresses Akt and PI3K phosphorylation, effectively blocking the PI3K/Akt signaling pathway activation in OV cell lines (37). Additionally, TRAF4 overexpression has been implicated in prostate cancer, where it mediates K27-linked ubiquitination of the AR C-terminus, elevates intracellular cAMP levels, enhances E2F transcription factor activity, and promotes cell proliferation (38).

UBE2L3, an E2 ubiquitin-conjugating enzyme, has been shown to reduce HPV16 E7 protein levels and inhibit tumor growth in HPV+ HNC cells through its overexpression (39). Similarly, UBE2L6 (aka UbcH8) is an essential ubiquitin-conjugating enzyme that controls the degradation of c-Myc through E3 ubiquitin ligases, thus regulating cell growth (40). UBE2L6 enhances the binding of ISG15 to cellular proteins and promotes apoptosis in cervical cancer cells (41). It has been identified as both a tumor suppressor and a prognostic marker for melanoma (42). Additionally, studies have demonstrated reduced expression of UBE2L6 in primary acute myeloid leukemia (AML) cells, where silencing UBE2L6 inhibits ATRA-induced ISG15 conjugation, thus impairing isgylation and hindering AML cell differentiation (43). In a study of 92 clinical samples from patients diagnosed with serous OV, immunohistochemical analysis showed marked correlation among UBE2L6 expression and platinum sensitivity. Given that UBE2L6 is implicated in platinum resistance (44), further *in vitro* and *in vivo* validation is warranted. The role of UBE2L6 in OV remains to be more thoroughly investigated in future studies. Moreover, the gene functions of the core genes FBXO45, UBE2L3, FBXL14, CHAF1B, and WDR77 in OV have yet to be explored, and this represents a promising avenue for further research.

FBXO45 is a constituent of the F-box family of proteins, which are a subfamily of the E3 ligase substrate recognition family (45). It has been reported that FBXO45 regulates malignant behaviors such as cell proliferation, metastasis, and drug resistance by ubiquitinating and degrading FBXW7 (46), and ZEB1 (47), but its function in ovarian cancer has not been reported. This study identifies FBXO45 as a potentially significant prognostic factor in OV, based on its highest single-factor regression coefficient among the core proteins (HR 1.0615). Single-cell sequencing results indicate that FBXO45 is most significantly associated with oocytes in ovarian fine. Recent studies have highlighted that oocyte depletion accelerates ovarian aging, which, in turn, contributes to cancer progression (48). It was also found that FBXO45 was highly expressed in ovarian plasmacytoid, mucinous and endometrioid cancer samples, and elevated levels of the protein expression were associated with poor prognosis. To validate the model’s predictive value, FBXO45 was silenced in A2780 and HEY cells, resulting in reduced cell proliferation, migration, and invasion. It is well known that the WNT signaling pathway regulates several key biological processes (cell proliferation, epithelial-mesenchymal transition, DNA damage response and chemotherapy tolerance) (49–51). Analysis of RNAseq data from OV patients in the TCGA database, coupled with GEPIA database and clinical patient sample analysis, further suggested that FBXO45 may enhance WNT/β-catenin signaling, thereby promoting the malignant phenotype of OV cells.FBXO45 frequently forms SCF complexes with Skp1 and Cul1 to perform its E3 ligase function (52), while FBXW7 is a substrate for ubiquitination degradation of FBXO45 (46). It has been found that FBXW7 inhibits TNBC cell stemness by ubiquitination degradation of the CHD4 protein. It has been established that the aforementioned mechanism functions by obstructing the activation of the Wnt/β-catenin pathway (53), Furthermore, research has demonstrated that the knockdown of SKP1 results in the inhibition of the Wnt signaling pathway, whilst concurrently inducing ROS production (54). The above literature further supports our conclusion.

OV is an immunogenic inflammatory disease closely associated with immune cell activity (55). Clinical trials have reported response rates to PD-1 and PD-L1 inhibitors in OV patients ranging from 4% to 15% (56). In our study, FBXO45 expression was positively correlated with naive B cells and M0 macrophages, while negatively correlating with the dormancy of monocytes and bone marrow dendritic cells. Additionally, FBXO45 expression was positive associated with the immune checkpoints CD274 (PD-L1). These suggests that it may promote the formation of an immunosuppressive microenvironment by inhibiting the antitumor activity of the host immune system. Meanwhile, tumor cells highly express PD-L1 and evade immune attack, thus promoting tumor growth and metastasis. These findings suggest that patients with higher FBXO45 expression may be more likely to respond positively to therapy involving PD-1 or PD-L1 inhibitors.

Our ubiquitination-related marker offers greater predictive value than other prognostic markers identified in previous studies, owing to the strong potential of ubiquitination-related factors for drug development. As an example, the UBE2L3 in prognostic model, whose small molecule inhibitor BAY 11–7082 has been shown to inhibit the inflammatory response, has been widely used (57). The pharmacological inhibition of TRAF4 by risperidone has been demonstrated to be an effective means of inhibiting tumor self-renewal in glioblastoma, with a concomitant reversal of temozolomide (TMZ) resistance (58).

As reported in previous articles, FBXO45 has been demonstrated to have pro-tumorigenic effects in cases of pancreatic, esophageal and lung cancers. Moreover, treatment of FBXO45-silenced lung cancer patients with afatinib has been shown to greatly increase patient sensitivity (45, 52, 59). In this study, FBXO45 was confirmed as a significant oncogene in OV, with its mechanism of action elucidated *in vitro*. This finding indicates that FBXO45 may represent a promising therapeutic target and that its clinical translation could prove advantageous for patients with diverse tumor types.

However, it should be noted that the study has limitations. Despite the study’s emphasis on the tumorigenic role of FBXO45 in OV, it is noteworthy that it lacked *in vivo* experiments and large-sample clinical trials. In order to understand the molecular mechanism more comprehensively, further 3D protein structure modeling (https://www.genecards.org/cgi-bin/carddisp.pl?gene=FBXO45#domains_families) (**Supplementary Figures S4**), structural domain identification, and proteomic screening of interacting proteins are needed to further analyze the pro-cancer mechanism of FBXO45 in depth. In addition, further research is required in the form of *in vivo* animal experiments, broader prospective clinical trials and larger sample studies in order to further explore the accuracy of prognostic models and the prognostic value of the key factor FBOX45 in ovarian cancer. Ultimately, this research will lead to clinical translation through the study of small molecule inhibitors and PROTACs.

## 5 Conclusion

Ubiquitination-related genes serve as reliable prognostic markers for OV and may inform clinical decision-making in patient management. As a core gene in the prognostic model, FBXO45 has the potential to function as a therapeutic target for ovarian cancer. Moreover, it can be argued that the results of this study provide a new concept for future targeted therapy against the Wnt signaling pathway.

## Data Availability

The original contributions presented in the study are included in the article/**Supplementary Material**. Further inquiries can be directed to the corresponding authors.

## Supplementary material

Supplementary Table 1 Enrichment analysis statistics.

Supplementary Figure 1 study scheme.

Supplementary Figure 2 Calibration plot illustrating the nomogram for predicting 1-, 3-, and 5-year OS.

Supplementary Figure 3 Identification of mutational features of the genome. **(A)** Waterfall plots of SNV data for the high-risk group. **(B)** Waterfall plots of SNV data for the low-risk group.

Supplementary Figure 4 3D structure of FBXO45 protein.
