# Supplementary figures and images for: Prognostic model of ubiquitination-related genes in ovarian cancer based on transcriptomic analysis and experimental validation
Source: Front Immunol. 2025 Sep 8;16:1654180. doi: 10.3389/fimmu.2025.1654180 (PMC12450667; doi:10.3389/fimmu.2025.1654180)

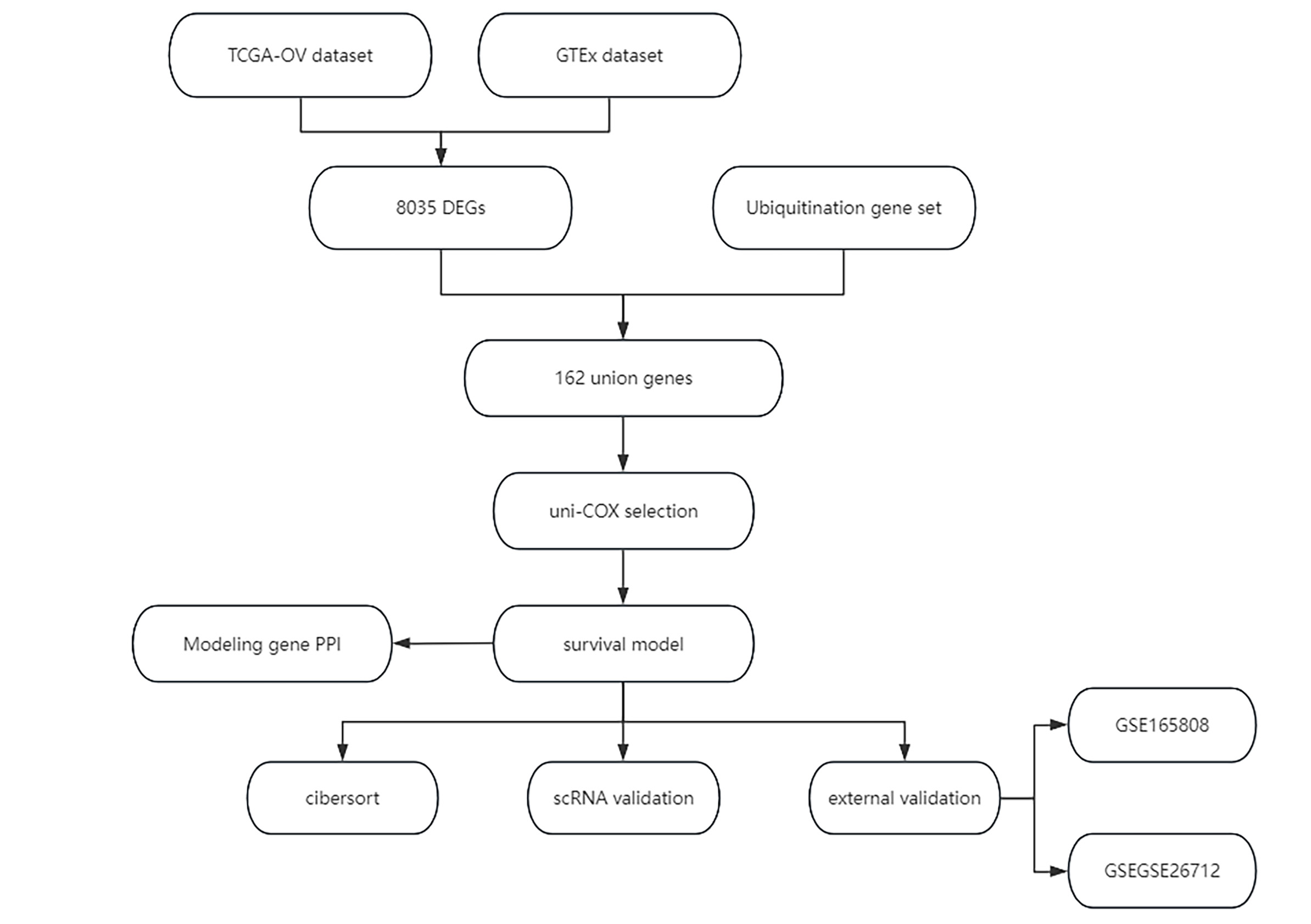

Supplement: Supplementary Figure 1 — study scheme. [file Image1.jpeg]

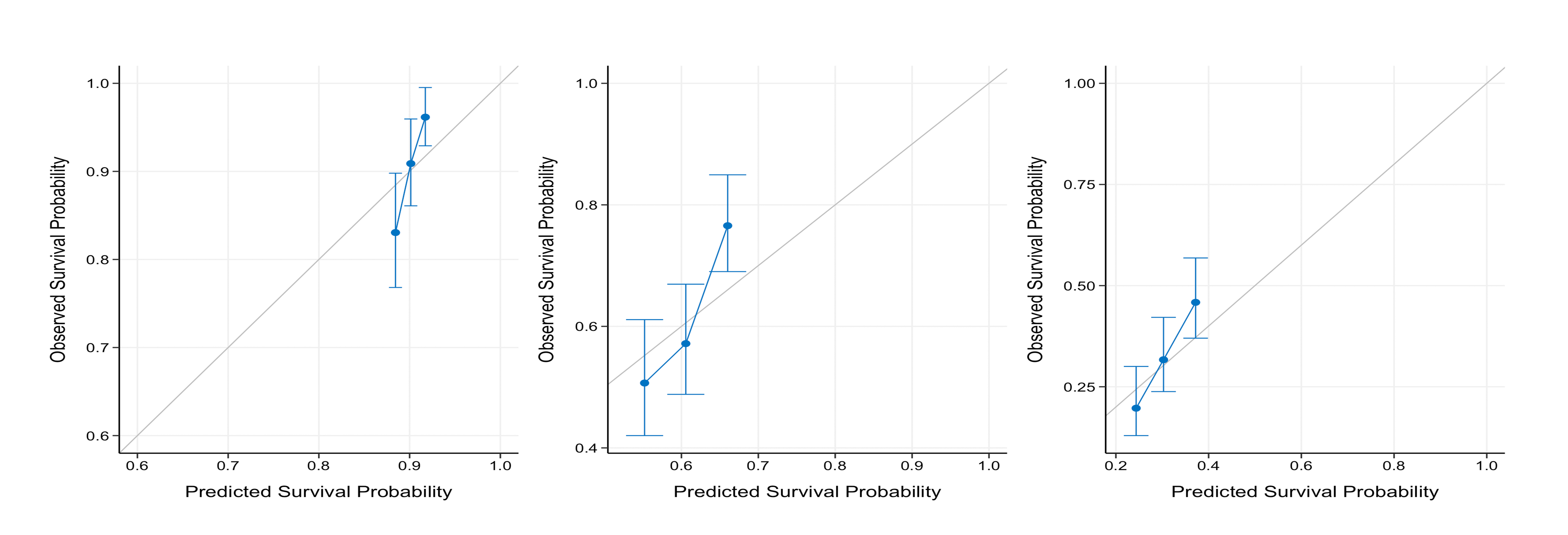

Supplement: Supplementary Figure 2 — Calibration plot illustrating the nomogram for predicting 1-, 3-, and 5-year OS. [file Image2.tif]

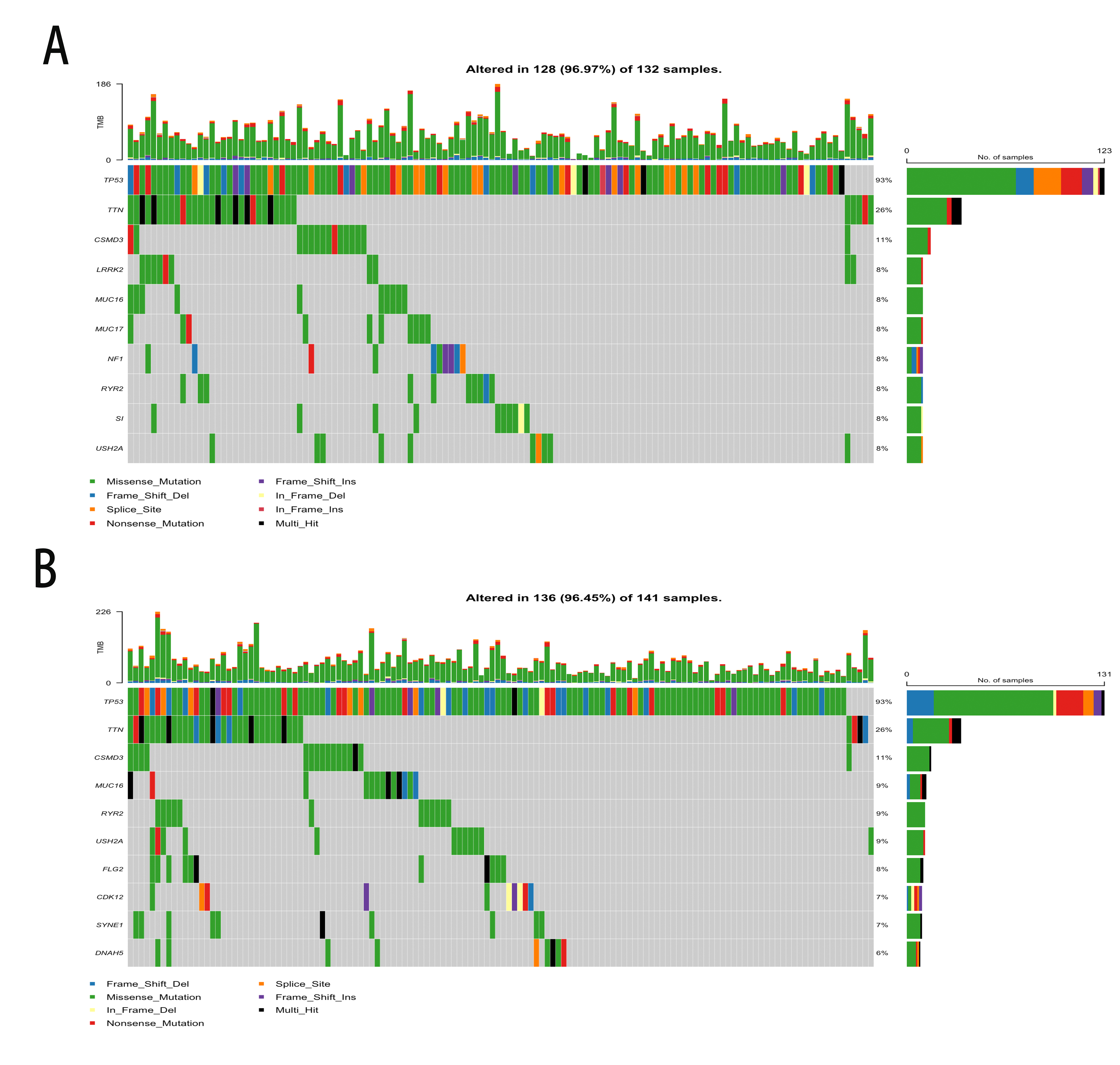

Supplement: Supplementary Figure 3 — Identification of mutational features of the genome. (A) Waterfall plots of SNV data for the high-risk group. (B) Waterfall plots of SNV data for the low-risk group. [file Image3.tif]

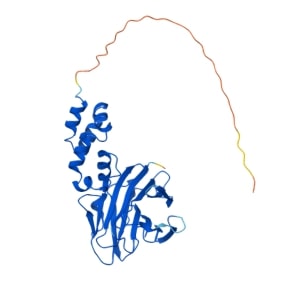

Supplement: Supplementary Figure 4 — 3D structure of FBXO45 protein. [file Image4.jpeg]
