# Supplementary material for: Prognostic model of ubiquitination-related genes in ovarian cancer based on transcriptomic analysis and experimental validation
Source: Front Immunol. 2025 Sep 8;16:1654180. doi: 10.3389/fimmu.2025.1654180 (PMC12450667; doi:10.3389/fimmu.2025.1654180)
Supplement: Supplementary file 6 [file DataSheet1.docx]

Materials and Methods

## Quantitative PCR

We followed the methods of Huang et al (1).qRT-PCR was used to assess mRNA expression. Total RNA was isolated from OV cells using Trizol reagent and reverse transcribed. Quantitative PCR involved 2 µg of mRNA, primers and SYBR Premix Ex Taq II. All materials were obtained from Takara (Japan). FBXO45 expression was normalised to GAPDH and quantified using the 2^(-∆∆Ct) method. Primers were sourced from Sangon Biotech, with the sequences detailed in Table 3

1.2 Design of qPCR primers:：

## The primer sequences under discussion were designed and supplied by Shanghai Bioengineering, a professional bioengineering company. Prior to utilisation, the sequences of the target primers were compared with the sequences of the gene in the human genome database using the BLAST tool to ensure the uniqueness of the product. Thereafter, the software Primer3 was employed to evaluate the following: the possibility of primer dimer formation（ΔG > -5 kcal/mol）、The formation of a hairpin structure（ΔG > -3 kcal/mol）、Self-complementarity（ΔG > -5 kcal/mol）、Tm between 55 and 65°C、The guanine-cytosine (GC) content must be between 40 and 60% The dimensions of the product were confirmed to be as expected by agarose gel electrophoresis, and finally the specific amplification was indicated by a PCR lysis curve to ascertain the presence of a single peak shape.

## 1.3 Establishment of predictive nomogram

A nomogram was created using the "hdnom" (version 6.0.2) (2) package to predict the ubiquitination-related risk score and clinical characteristics of OV. Calibration plots compared the predictions of one-year, three-year and five-year survival probabilities with real outcomes, validating the model's consistency across different clinical and pathological parameters.

1.4 scratching test

Ovarian cancer (A2780, HEY) cells in logarithmic growth phase were resuspended and evenly inoculated in 6-well plates. When the cell density reached approximately 90%, a 200 μL sterile lance tip was used to make scratches in the well plates and the cells were gently rinsed with PBS. Subsequently, 2 mL of serum-free medium was added to continue the culture. Photographs were taken to record the scratched area of the cells immediately at 0 h after scratching. Later, photos were taken again at 24 and 48 hours after scratching to record cell migration, with 3 fields of view per well. Using ImageJ software (steps: grayscaling→threshold segmentation→measurement of scratch area), the migration rate (%) = (initial area - end area) / initial area × 100%, the scratch area at each time point was calculated, and independent experiments were performed for 3 times, and t-tests were performed using GraphPad Prism to assess the cell migration ability.

1.5 Migration and invasion

Cells in the logarithmic growth phase were starved overnight with serum-free medium to remove the effects of growth factors and other nutrients from the serum. The supernatant was discarded the following day and washed twice with PBS. Cells were digested with 0.25% trypsin, collected and centrifuged at 1200 rpm for 5 min, and the supernatant was discarded. Cells were resuspended using 1 mL of serum-free medium and cell counting was performed. Based on the counting results, A2780 cells were adjusted to 5 × 10⁴ cells/well and HEY cells were adjusted to 6 × 10⁴ cells/well. Add 600 μL of medium containing 10% fetal bovine serum (FBS) to the lower chamber of the 24-well plate. The Transwell was carefully placed into the 24-well plate. The cell suspension with adjusted concentration was carefully added to the upper chamber of the Transwell at 200 μL/well to avoid air bubbles. After the cells were incubated in an incubator at 37°C and 5% CO₂ for 48 h, the Transwell chambers were washed twice with PBS to remove residual medium and non-invasive cells, and after the chambers were washed twice with PBS again, they were fixed using 4% paraformaldehyde for 30 min, and after fixation was completed, the chambers were washed three times with PBS, and stained using 0.3% crystal violet solution for 15 min. The chambers were washed three times with PBS, and the uninvaded cells in the upper layer of the Transwell were gently wiped away with a cotton swab. The chambers were again washed with PBS to ensure that all residual cells were removed, and the chambers were placed at room temperature to air-dry. After air-drying, five randomly selected fields of view/membrane (20× objective) were photographed by microscope, and the number of infiltrating cells was manually counted independently by two experimentalists using ImageJ. The independent experiments were performed three times. For the number of migrating/invading cells, a t-test was performed using GraphPad Prism.The invasion experiments were performed in advance using Matrigel (1:9 dilution) coated with Transwell membrane (overnight at 4°C), and the rest of the steps were the same as the migration experiments.

1.6 CCK8

After the cells entered the logarithmic growth phase, the original medium was discarded and washed twice with PBS. Subsequently, cells were treated with 0.25% trypsin and centrifuged at 1000 rpm/min for 5 min to collect the cell precipitate. Cells were resuspended by adding 1 mL of complete medium and counted using Oxbow counting plates. 200 μL of cell suspension containing 5×103 cells was added into 96-well plates, with at least 3 replicate wells for each experimental group, and the edge wells were filled with PBS to minimize the evaporation effect, and the plates were placed into the incubator for 72 h. The original medium in the well plates was carefully removed at 0 h, 24 h, 48 h, and 72 h, respectively, and 100 μL of serum-free medium containing 10% CCK-8 reagent was added to each well. The well plates were incubated in an incubator at 37℃ for 2 hours to ensure that the CCK-8 reagent fully reacted with the cells, and the optical density (OD) of each well was measured at 450 nm using an enzyme marker. Independent experiments were performed three times, t-tests were performed using GraphPad Prism, and data were expressed as mean ± SEM.

Table3. Gene-specific primers for qRT-PCR.

| Gene | Forward (5’-3‘) | Reverse (3’-5‘) |
| --- | --- | --- |
| FBXO45 | CAAGGCCAAGATACGTGCTTT | GAGCAATGGGGTTTCGATGTA |
| GAPDH | GGAGCGAGATCCCTCCAAAAT | GGCTGTTGTCATACTTCTCATGG |

References

1. Huang Y, Li L, Kang Z, Luo H, Lin X, Zhao S, et al. Prognostic Model Associated with Necroptosis in Colorectal Cancer based on Transcriptomic Analysis and Experimental Validation. FBL. 2024;29(3).

2. Wadasadawala T, Kannan S, Gudi S, Rishi A, Budrukkar A, Parmar V, et al. Predicting loco-regional recurrence risk in T1, T2 breast cancer with 1-3 positive axillary nodes postmastectomy: Development of a predictive nomogram. Indian J Cancer. 2017;54(1):352-7.
